# The Impact of FFC-AL-EIT on Resident and Setting Outcomes

**DOI:** 10.1093/geroni/igab046.1323

**Published:** 2021-12-17

**Authors:** Marie Boltz

**Affiliations:** Pennsylvania State University, University Park, Pennsylvania, United States

## Abstract

FFC-AL-EIT was implemented by a Research Nurse Facilitator working with a community champion and stakeholder team for 12 months to increase function and physical activity among residents. FFC-AL-EIT included four steps: (Step I) Environment and Policy Assessments; (Step II) Education; (Step III) Establishing Resident Function Focused Care Service Plans; and (Step IV) Mentoring and Motivating. A total of 85 communities and 794 residents were included. The age of participants was 89.48 (SD=7.43), the majority was female (N=561, 71%) and white (N=771, 97%). Resident measures, obtained at baseline, four and 12 months, included function, physical activity, and performance of function focused care. Setting outcomes, obtained at baseline and 12 months, included environment and policy assessments and service plans. Effectiveness was based on less decline in function (p<.001), more function focused care (p=.012) and better environment (p=.032) and policy (p=.003) support for function focused care in treatment sites.

